# The Spectrum of Disease Manifestations in Patients with Common Variable Immunodeficiency Disorders and Partial Antibody Deficiency in a University Hospital

**DOI:** 10.1007/s10875-012-9671-6

**Published:** 2012-04-13

**Authors:** L. J. Maarschalk-Ellerbroek, A. I. M. Hoepelman, J. M. van Montfrans, P. M. Ellerbroek

**Affiliations:** 1 Department of Internal Medicine and Infectious Diseases, University Medical Centre Utrecht, P.O. Box 85500, 3508 GA Utrecht, the Netherlands; 2 Departments of Paediatric Immunology and Infectious Diseases, University Medical Centre Utrecht/Wilhelmina Children’s Hospital, Utrecht, the Netherlands

**Keywords:** Common variable immunodeficiency, clinical manifestations, diagnostic delay, pulmonary disease, lymphoproliverative, gastrointestinal disease, autoimmune disease, malignancy, B lymphocyte, T lymphocyte, flow cytometry

## Abstract

**Background:**

Common variable immunodeficiency disorders (CVIDs) represents a heterogeneous disease spectrum that includes recurrent infections and complications such as autoimmunity, inflammatory organ disease and an increased risk of cancer. A diagnostic delay is common in CVIDs patients.

**Purpose:**

To determine the spectrum of clinical manifestations, immunological characteristics, and the time to diagnosis of 61 adult CVIDs and 18 patients with a partial antibody deficiency (SADNI and IgG subclass deficiency).

**Methods:**

A retrospective cohort study was performed in patients who met the ESID/PAGID for CVIDs, IgG subclass deficiency and SADNI. Medical records were reviewed to obtain patient demographics, clinical and laboratory data.

**Results:**

Infections were the main presentation of all antibody deficient patients and the number of patients with infections declined during IgG therapy. The development of bronchiectasis continued despite IgG therapy, as well as the development of autoinflammatory conditions. Non-infectious disease complications were present in 30% of CVIDs patients at the time of diagnosis and this increased to 51% during follow up despite IgG therapy. The most common complications were autoimmunity or lymphoproliferative disease. The median time to diagnosis was 10 years and in the patients with non-infectious complications the time to diagnosis was considerably longer when compared to the group of patients without complications (17.6 vs. 10.2 years, *p* = 0.026).

**Conclusion:**

In contrast to the partial antibody deficiencies we found a considerable delay in the diagnosis of CVIDs, especially in those patients who were dominated by non-infectious complications, and thus increased awareness would be beneficial. Pulmonary and other complications may continue despite adequate IgG replacement therapy suggesting other causes responsible for these complications.

**Electronic supplementary material:**

The online version of this article (doi:10.1007/s10875-012-9671-6) contains supplementary material, which is available to authorized users.

## Introduction

Primary antibody deficiencies represent the largest group of primary immunodeficiencies and are characterized by B-cell dysfunction [1]. Common variable immunodeficiency disorders (CVIDs) is the most heterogeneous group among the antibody deficiency syndromes and the estimated prevalence is 1: 25.000 [2]. The typical clinical presentation of CVIDs is that of recurrent bacterial respiratory tract infections but other infections may also occur. Previous gene defects have found to be associated with the CVIDs phenotype (ICOS, TACI, CD19, BAFF-R, MSH5, CD20 and CD81) [3–5], however, less than 10% presents within families. In most patients symptoms will start after puberty, and the diagnosis is often made when the patient has already reached adulthood. A diagnostic delay is quite common with a mean of 6–8 years after the onset of symptoms [6–8], it can however take longer than a decade before a patient is diagnosed [9]. The diagnostic delay and subsequent delay in (immunoglobulin) therapy is thought to be a major cause of the development of organ damage resulting in increased morbidity and mortality [8–12]. Pulmonary damage is the most frequent complication and may result from recurrent infections and/or immune dysregulation. Other complications due to the underlying immune dysregulation [7, 9, 13] include lymphoproliferative disease (granulomatous disease [14], lymphadenopathy and hepatosplenomegaly), autoimmune disease [15], gastrointestinal disease such as chronic inflammation [16] and an increased risk of cancer [17, 18]. While a substantial part of the CVIDs patients remain relatively free of such problems, a subgroup will develop one or more of these disease related complications. Attempts have been made to classify the heterogeneous CVIDs population into more similar subgroups by using clinical parameters [9] and flow cytrometric markers of B- [13, 19, 20] and T cells [21, 22] in order to define parameters that might predict which patients will be particularly prone to these complications.

Clinically less severe and much more common antibody deficiencies are IgG subclass deficiency and Selective antibody deficiency with normal immunoglobulins (SADNI) that are mainly characterized by recurrent sinopulmonary infections. In 20% of cases however, the finding of lower IgG subclass levels is merely a laboratory finding that does not necessarily lead to symptoms [23]. Nevertheless, these conditions occasionally progress to CVIDs [23, 24]. In the current retrospective study we determined the spectrum of clinical manifestations during the long-term follow-up of adult CVIDs patients in the outpatient clinic of our university hospital. For comparison we also included patients with an IgG subclass deficiency or SADNI. We studied the clinical features, the immunological characteristics and the time to diagnosis according to the previous presented classification systems [9, 13].

## Methods

The department of internal medicine and Infectious diseases at the University Medical Centre Utrecht in The Netherlands serves as a referral center for adult patients with primary immunodeficiencies. We performed a retrospective cohort study of all patients with an antibody deficiency that met the ESID/Pan-American Group criteria for Immunodeficiency (PAGID) [25] for Common Variable Immunodeficiency disorders (CVIDs), IgG- subclass deficiency and Selective antibody deficiency with normal immunoglobulins (SADNI) that have attended our outpatient clinic between 1978 and 2011. Selective antibody deficiency with normal immunoglobulins was defined as a failure to produce antibodies to polysaccharide vaccines. The response to 23-valent pneumococcal polysaccharide vaccine was assessed 4 to 6 weeks after vaccination and evaluated according to age and vaccination history. If the adult patient was not previously vaccinated with a conjugated pneumococcal vaccine, the IgG response to the 23 valent polysaccharide vaccine (Pneu23) was found abnormal when less than 8 more of the 11 measured antibody titres had reached a value of ≥1.0 μg/ml.

Patients with a hyper IgM syndrome, XLA or congenital agammaglobulinaemia were excluded from this study because of the distinct entity and so where patients with a secondary hypogammaglobulinaemia due to protein loss, drugs, malignancy, or infection. All data entries were crosschecked by an independent physician

### Clinical Follow Up

Medical records from the patients were reviewed to obtain patient demographics, clinical manifestations and laboratory data. Clinically stable patients had usually visited with a frequency of once or twice a year at our outpatient clinic. Routine physical examination together with standard laboratory measurements and IgG trough levels were performed in CVIDs patients once or twice a year. Until recently a pulmonary function test, High Resolution Computed Tomography (HrCT), abdominal ultrasound and a gastro- and colonoscopy were performed only in case of symptoms, this policy has changes the last 2 years.

The time to diagnosis was defined as the time in years between the year of onset of disease-related symptoms (infectious or non-infectious complications as depicted below) and the year of diagnosis. Patients that still had ongoing infections after the start of IgG therapy were scored when antibiotics were prescribed or when positive bacterial culture results were obtained in combination with a clinical diagnosis. To assess the onset of disease we used the following clinical criteria based on the ESID PID warning signs: 1) ≥4 new middle ear infections within 1 year. 2) ≥2 or more sinus infections within 1 year. 3) ≥2 months on antibiotics with little effect. 4) Two or more pneumonias within 1 year or recurrent pneumonias. 5) Recurrent, deep skin or organ abscesses. 6) Persistent thrush in mouth or fungal infection on skin. 7) Need for intravenous antibiotics to clear infections. 8) Two or more deep-seated infections including septicaemia (i.e. osteomyelitis, meningitis, severe pneumonia, and arthritis). 9) Gastrointestinal infections with Giardia Lamblia or Campylobacter.

All immunologic data was entered twice in the database by different persons.

### Disease Complications

Symptomatic chronic pulmonary disease (CPD) was defined as chronic obstructive pulmonary disease or asthma, bronchiectasis or inflammatory pulmonary conditions (such as interstitial lung disease). Other complications related to immune dysregulation have been previously categorized into five phenotype categories [9]: (1)lymphoproliferative disease, (2) autoimmune disease, (3) gastrointestinal disease (4) malignancies or (5) no disease related complications. Patients were scored according to these categories (i.e. 1 complication = 1 category) and individual patients were scored for having one or more complications.

Autoimmunity included cytopenias (chronic autoimmune hemolytic anemia, chronic autoimmune thrombocytopenia, and unexplained leucocytopenia) and organ-specific autoimmunity (rheumatoid arthritis and systemic lupus erythematosus meeting the American Rheumatism Association [ARA] criteria, Graves’ disease, pernicious anemia and atrophic gastritis (biopsy proven) and alopecia areata). Lymphoproliferative conditions were defined as unexplained persistent lymphadenopathy (on palpitation, ultrasound or computer tomography scan), granulomatous disease (biopsy proven) or hepatosplenomegaly (ultrasound proven).

Gastrointestinal disease was defined as gastrointestinal symptoms combined with biopsy proven endoscopic abnormalities and included Helicobacter pylori positive gastritis, inflammatory colitis, malabsorption with villous atrophy, polyps and adenoma. Malignancies were defined as biopsy proven lymphoid, bone marrow or solid organ cancer.

### Laboratory Data

Immunoglobulin titres, T and B cell phenotyping and in vitro mitogenic and antigenic T cell proliferation responses had been performed in most patients, between 2007 and 2011. The timing was aimed just before the administration of immunoglobulins. At the time of these measurements only one patient used prednisolone 10 mg/day on a chronic basis. IgG trough levels had been measured once or twice a year in clinically stable patients during follow up, and more often in patients with disease-related complications. In our current daily practice we aim to reach IgG through levels of at least 8 g/L [26].

CVIDs patients were classified according to the classification of EURO Class trial [13], a classification scheme based on flowcytometric B-cell phenotyping and the clinical course of the patient. The T and B cell populations were analyzed by four-color flow cytometry using whole blood and antibodies to CD3, CD45, CD27, CD4, CD8, HLA-DR, CD38, CD45RA and CD19, CD27, CD38, CD10, IgM, IgG, IgA, IgD, respectively, as described previously [27, 28].

For the T and B lymphocyte functional assays peripheral blood mononuclear cells (PBMC) were obtained and the following stimuli were supplemented: phytohemagglutinin, Concanavalin A, tetanus toxoid, purified protein derivative (PPD), Candida albicans and diphtheria toxin. Assay conditions were verified by a control sample run in parallel. The percentage of response was defined by the number of positive responses to a stimulus divided by the total number of tests. For B cell differentiation assays, PBMC were cultured with either pokeweed mitogen or Staphylococcus aureus antigen and IL-2 [28].

### Statistical Analysis

Statistical analyses were performed using Mann–Whitney U tests and Pearson’s chi square tests with SPSS 15.0 for Windows. A P value of 0.05 or less was considered significant.

## Results

Sixty-one CVIDs patients, nine IgG subclass deficiency patients and nine patients with Selective antibody deficiency with normal immunoglobulins (SADNI) were analyzed. All patients had been diagnosed between 1978 and 2010. The age at onset of symptoms could be traced in 55 of 61 CVIDs patients, the year of diagnosis was known for all patients.

### Common Variable Immunodeficiency

The baseline characteristics are shown in Table I. The median age of the 58 CVIDs patients that were still alive at the time of analysis was 38 years (IQR 26–58 years). Of all patients that were analyzed 36 were female (59%) and 25 male (41%) and the vast majority of patients were Caucasian (56 patients, 92%). The recorded follow up in our hospital since diagnosis ranged between 4 and 13 years (median 7 years). The median age at which CVIDs related symptoms had started was 17 years (IQR 4–23 years) and the median age at diagnosis had been 27 years (IQR 14–43 years). The median time to diagnosis had been 10 years (IQR 5–16 years), which is addressed in further detail below.

**Table I:** Baseline characteristics

	CVIDs	IgG subclass deficiency	SADNI
	(*n* = 61)	(*n* = 9)	(*n* = 9)
Sex, number of pt (%)			
female	36 (59%)	7 (78%)	6 (67%)
male	25 (41%)	2(22%)	3 (33%)
Ethnicity, number of pt (%)			
Caucasian	56 (92%)	8 (89%)	9 (100%)
middle East	2 (3%)		
Far east	1 (2%)	1 (11%)	
mix	2 (3%)		
Current age, median (IQR)	38 (26–58) yrs	42 (25–49) yrs	45 (26–53) yrs
Age at start symptoms, median (IQR)	17 (4–23) yrs	16 (4–38) yrs	33 (20–45) yrs
Age at diagnosis, median (IQR)	27 (14–43) yrs	38 (14–45) yrs	44 (21–52) yrs
Time to diagnosis, median (IQR)	10 (5–16) yrs	4 (1–24) yrs	2 (1–10) yrs
Follow up since diagnosis, median (IQR)	7 (4–13) yrs	3 (1–12) yrs	1 (1–3) yrs
Follow up since start therapy, median (IQR)	6,5 (3–13) yrs	3 (1–11) yrs	1 (0–2,5) yrs
Death, number of pt	3	0	0
Causes of death	pneumonia, brain abscess sepsis with pneumonia		
Number of patients with family members with a confirmed antibody deficiency	6 (in 3 families)	0	0
Number of pt with renal failure	3	0	0

*Pt* patients, *CVIDs* common variable immunodeficiency disorders, *SADNI* selective antibody deficiency with normal immunoglobulins. ^a^First or second degree family members

**Fig. 1:**
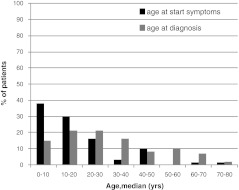
Age at onset symptoms and at diagnosis of CVIDs in retrospective analysis

The majority of CVIDs patients (42 patients, 69%) already had related symptoms before the age of 20 years, however, only 36% had been diagnosed before the age of 20 suggesting a substantial time to diagnosis (Fig. 1). Notably, two patients had developed symptoms after the age of 60 years.

In all CVIDs patients intravenous (*n* = 45) or subcutaneous (*n* = 16) IgG substitution therapy had been started. The vast majority had commenced this therapy within 1 year after the diagnosis had been established (54 patients, 88%). All patients had been started on immunoglobulin therapy in our hospital, either in the department of pediatrics or in the adult department and follow up of patients took place in our hospital. Currently, it is standard to start with immunoglobulin dosing of 0.4 g/kg. The dosage of patients differs and had been adjusted according to IgG trough levels (aim > 8.5 g/l) and clinical response. The mean IgG trough level in the last decade was 8.8 g/L. Twenty-six patients (43%) also received antibiotic prophylaxis at any point during follow up.

#### Infections

Before immunoglobulin therapy 90% (*n* = 55) of CVIDs patients had suffered from (recurrent) respiratory infections as shown in Table II. Four patients (7%) had suffered from severe herpes virus infections (Varicella and/or Herpes simplex) before diagnosis. Infections of the urogenital tract, central nervous system, gastro-intestinal tract and skin were much less common. Four patients (7%) had no recurrent or severe infections before the diagnosis and this remained so thereafter: two patients had arthralgia at the time, one was screened because of a CVIDs sibling, and the last patient was screened for chronic non-infectious diarrhea.

**Table II:** The number of patients (%) with infections prior to diagnosis

	CVIDs	IgG subclass deficiency	SADNI
	(*n* = 61)	(*n* = 9)	(*n* = 9)
	Number of patients (%)	Number of patients (%)	Number of patients (%)
None	4 (7%)	0	0
Recurrent respiratory infections	56 (93%)	9 (100%)	9 (100%)
URTI	43 (71%)	3 (33%)	6 (67%)
LRTI	31 (51%)	6 (67%)	5 (56%)
Gastrointestinal infection¹	7 (12%)	0	0
Recurrent	6/7 (86%)	NA	NA
Other infections *(number of pt)*			
Abdominal abcess	1		1
Skin infections	3	1	
Herpes virus	4		
Hepatitis	1		
Meningitis	3	2	1
Pancarditis	1		
UTR	5	1	
Panuveitis (toxoplasma)	1		

*Pt* patients, *CVIDs* common variable immunodeficiency disorders, *SADNI* selective antibody deficiency with normal immunoglobulins. ^a^Gastrointestinal infections: Giardia Lambliae, Campylobacter enteritis, Salmonella enteritis

**Fig. 2:**
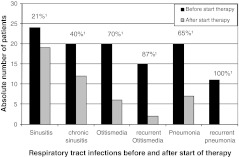
Number of CVIDs patients with respiratory tract infections before and after start of immunoglobulin therapy. ¹% decline number of patients with respiratory tract infections

The median IgG trough level of the patients with infections after start of IgG therapy was not significantly different in comparison to the patients without infections (9.2 g/L vs. 8.7 g/L, respectively). Although eight of the 55 patients (14%) with respiratory infections became free of infections after the initiation of IgG therapy the majority of patients still suffered from respiratory infections (47 of 55 patients, 85%; Table II), although these appeared to be less frequent. Figure 2 shows the reduction in the number of patients with respiratory tract infections following the institution of immunoglobulin therapy. The most prominent reduction was established in middle ear infections and pneumonia (70–100% reduction; Fig. 2). However, least effect was accomplished in the occurrence of sinusitis: 79% of patients with sinusitis prior to IgG therapy still suffered from one or more episodes and 60% of patients with chronic sinusitis were not cured.

Seven (11%) patients had suffered from gastrointestinal infections before diagnosis of which 4 with Giardia Lamblia, eight more had a gastrointestinal infection (13%) after start of therapy. During follow up one patient was diagnosed with Progressive Multifocal Leukoencephalopathy (PML) during prednisolone treatment for interstitial pulmonary disease and one patient with CMV colitis.

#### Pulmonary Disease and Chronic Sinusitis

Symptomatic chronic pulmonary diseases (CPD) was diagnosed in 20 (33%) CVIDs patients before the start of therapy and this number increased to 34 (56%) patients after the start of immunoglobulin therapy (Table III). Before the start of therapy the majority had been diagnosed with asthma (13 of 20 patients) and none during follow-up. Chest CT scanning demonstrated the presence of bronchiectasis in two patients at diagnosis and in another eight during the follow-up, which is likely to be an underestimation since only 12 patients underwent chest CT scanning at or before diagnosis. Of the eight patients diagnosed with bronchiectasis during follow up only two patients had mean IgG trough levels <8 g/L. Another three patients developed interstitial lung disease during follow up. Chronic sinusitis was present in 20 patients (33%) and responded in eight patients to IgG therapy.

**Table III:** Symptomatic chronic lung disease in 61 CVIDs patients

	Before start of therapy	After start of therapy
	Total number of patients (%)	Total number of patients (%)
Symptomatic chronic pulmonary disease^a^	20/61 (33%)	34/61 (56%)
Structural airway changes (bronchiectasis)	2/20 (10%)	10/34 (29%)
Interstitial lung disease	6/20 (30%)	9/34 (26%)
Granulomatous disease	3/6 (50%)	3/9 (33%)
Fibrosis	3/6 (50%)	6/9 (67%)
COPD/asthma	13/20 (65%)	16/34 (47%)

^a^Symptomatic chronic lung disease was defined as chronic obstructive pulmonary disease or asthma, complications due to infections (e.g. bronchiectasis) and auto inflammatory pulmonary conditions such as interstitial lung disease

#### Other Disease Related Complications

Table IV displays the number and nature of CVIDs related complications and the different disease complications observed before diagnosis and during follow up. Despite immunoglobulin therapy patients still developed complications during follow up.

**Table IV:** CVIDs related complications before and after start of immunoglobulin therapy in 61 CVIDs patients

	Before start of therapy	After start of therapy
	Number of patients (%)	Number of patients (%)
*Complications*		
Yes	18 (30%)	31 (51%)
No	43 (70%)	30 (49%)
*Number of complications/patient*		
0	43 (70%)	30 (49%)
1	14 (23%)	18 (29,5%)
2	4 (6,5%)	9 (15%)
3	0	4 (6,5%)
4	0	0
*Type of disease (Number of patients)* ¹		
Lymphoproliverative	8/61 (13%)	17/61 (28%)
Granulomatous disease	4	8
Lymphadenopathy	4	11
Hepatosplenomegaly	4	11
Spleen	2	8
Liver	1	1
Both spleen and liver	1	2
Autoimmune disease	10/61 (16%)	14/61 (23%)
Non-septic arthritis	2	2
Autoimmune cytopenia	3^b^	9
Organ related	2^c^	3^c^
Alopecia	3	3
Malignancy	0	4/61 (7%)
Anal	0	1
Thyroid	0	1
Seminoma	0	1
Bladder	0	1
Gastrointestinal disease	4/61 (6,5%)	13/61 (21%)
Oesofagits		2
Gastritis	1	7
Villous atrophy	1	5
Inflammation ileum/colon/rectum	2	8
Angiodysplasy		1
Polyps/adenoma	1	4
Malignancy		1
Nodular lymphoid hyperplasia		6
*Number of patients with phenotypes*		
None	43 (70%)	30 (49%)
Lymphoproliverative	4 (7%)	5 (8%)
Autoimmune	9 (15%)	8 (13%)
gastrointestinal disease	1 (1.5%)	4 (6,5%)
Malignancy	0	1 (1,5%)
Lymphoproliverative and autoimmunity	1 (1.5%)	3 (5%)
Lymphoproliverative and gastrointestinal disease	3 (5%)	5 (8%)
Lymphoproliverative and malignancy	0	1 (1,5%)
Autoimmune, malignancy and gastrointestinal disease	0	1 (1,5%)
Lymphoproliverative, autoimmune and gastrointestinal disease	0	2 (3%)
Lymphoproliverative, malignancy and gastrointestinal disease	0	1 (1,5%)

^a^patients can have more than 1 condition

^b^Of the three patients with cytopenia, one male suffered from autoimmune hemolytic anemia and two females from Idiopathic thrombocytopenic purpura

^c^Before diagnosis of CVID: one patient was diagnosed with diabetes Mellitus and another patient with Systemic lupus erythematodes., After diagnosis one patient was diagnosed with hypothyroidism

**Table V:** Immunological parameters of CVIDs patients

	(*n* = 61)	Females (*n* = 36)	Males (*n* = 25)
*Ig levels at diagnosis (g/L)*	*Median (range)*	*Median (range)*	*Median (range)*
			
IgG (7,0–16,0)	4,0 (2,3–5,1)	4,0 (2,4–5,0)	3,9 (1,12–5,3)
IgA (0,7–4,0)	0,1 (0,0–0,6)	0,3 (0,0–0,66)	0,04 (0,2–0,42)
IgM (0,4–2,3)	0,4 (0,22–0,9)	0,5 (0,26–1,04)	0,31 (0,2–0,43)
*Number of patients (%) with lymphocyte below lower limit counts* ^*a*^	*Number of patients (%)*	*Median (range)*	*Number of patients with complications*
CD3^b^	10/52 (19,2%)	(376–684 cells/ul)	9/10
CD4^b^	12/54 (22,2%)	(55–344 cells/ul)	9/12
CD8^b^	5/52 (9.6%)	(37–155 cells/ul)	5/5
Inverted CD4/CD8 ratio^b^			
Under lower limit	10/53 (18.7%)	(0.30–0.90)	5/10
Above upper limit	7/53 (13.2%)	(3.5–9.0)	4/7

^a^absolute lymphocyte counts per cubic millimeter

^b^reference values: CD3 (100–400), CD4 (400–1300), CD8 (200–700), CD4/CD8 ratio (1,1–3,2)

At diagnosis 18 patients (29%) had one (14 patients) or more complications (4 patients) and this number increased to 31 patients (51%) during follow up. Of the 18 patients who already had one or more complication at diagnosis seven patients developed additional complications of a different etiology. Additional or new complications developed in 20 patients (33%) during follow-up. Of more than half of the patients that developed new complications during follow-up (18 of 31; 58%) these complications could be categorized into one single category. Lymphoproliferative and autoimmune complications were the most frequent complications that were already present at the time of diagnosis. Newly diagnosed complications during follow up were mostly of a lymphoproliferative or gastrointestinal nature. Splenomegaly was fairly uncommon in our cohort at diagnosis (only three patients at diagnosis and another six during follow-up) which is likely an underestimation since abdominal ultra sound was not performed routinely. None of the patients had been diagnosed with cancer before the diagnosis of CVIDs and during follow-up four patients developed a malignancy (anal carcinoma at the age of 27, thyroid cancer at the age of 22, seminoma at the age of 58 and bladder cancer at the age of 61). Three of these patients also had other complications as a result of extended immune dysregulation (Table IV). All three patients who developed end stage organ failure during follow up were diagnosed with terminal renal insufficiency and are currently on hemodialysis. In two patients renal insufficiency was caused by underlying vascular problems and one patient was diagnosed with interstitial nephritis.

The IgG trough levels of the patients that had developed new complications since IgG substitution did not differ significantly of that of patients that had not.

#### Time to Diagnosis

**Fig. 3:**
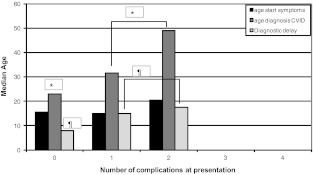
Median age at start of symptoms, at diagnosis and diagnostic delay of CVIDs patients compared to the number of CVIDs related non-infectious complications *at diagnosis.* * and ¶: patients without any complication vs. patients with one or more complication: *p* < 0.05

The median time to diagnosis of CVIDs was substantial, 10 years (IQR 5–16 years) compared to 2.5 years (IQR 1–18 years), *p* = 0.016 of the partial antibody deficiencies (IgG subclass deficiency and SADNI taken together).Two patients with bronchiectasis at diagnosis had a diagnostic delay as long as 24 and 32 years, respectively. Figure 3 shows the age at the onset of symptoms and the age at the time of CVIDs diagnosis as well as the time to diagnosis in relation to the number of complications. Patients with one or two complications had been significant older at the time the CVIDs diagnosis was made compared to the patients without complications. (*n* = 17 vs. 44, 39 year vs. 28 year, *p* = 0.03) The median time to diagnosis in the group of patients with non-infectious complications was seven years longer in comparison to the group of patients without these disease complications (10.2 vs. 17.6 years, *p* = 0.026). Patients with autoimmune disease (10 patients) had a median diagnostic delay of 17 years (IQR 0–39 years). Especially alopecia had been present long before the diagnosis of CVIDs was made (mean 25 years, range 11–39 years). All four patients with granulomatous disease had a relative long time to diagnosis (respectively 32, 21, 16 and 7 years). Of the four patients (6.5%) with gastrointestinal disease diagnosed before the diagnosis of CVIDs, the diagnostic delay was 32, 10, 7 and 5 years respectively. The diagnostic delay of the three patients that had died during follow up had been 28, 14 and 12 years, respectively.

#### Deaths

Four patients had died during follow-up. One male patient had been diagnosed with CVIDs 14 years after his symptoms started at the age of 63 years. He also suffered from cardiovascular disease and diabetes mellitus and died at the age of 70 years as the result of pneumonia. A female patient died at the age of 31 years due to a brain abscess. She had been diagnosed with CVIDs at the age of 27 after suffering from upper respiratory tract infections, Herpes Zoster infections and lymphoproliferative disease for 12 years (since the age of 15). The third patient, also a female, died at the age of 49 due to a sepsis of unknown cause. She had been diagnosed at the age of 45 years but had suffered from numerous clinical problems years before that (since the age of 17 years).

#### Laboratory Evaluation

The median IgG of all CVIDs patients at diagnosis was 3.8 g/L (IQR 2.1–4.9 g/L) (Table V).

Patients who were diagnosed with lymphoproliferative conditions, autoimmune disease and gastrointestinal disease had a lower IgG at diagnosis compared to those without complications. (2.0 g/L (IQR 1.2–3.6) *p* = 0.02; 2.8 g/L (IQR 1.6–4.4) *p* = 0.03; 1.5 g/L (IQR 0.63–2.9) *p* = 0.002 vs. 4.5 g/L (IQR 2.8–5.2) respectively).

#### B Cell Phenotype

During routine clinical evaluation flowcytometric B-cell phenotyping had been performed in 46 patients, and 70% of these patients had normal numbers of total CD19 positive B cells. Patients with complications related to immune dysregulation had lower absolute numbers of CD19 positive B lymphocytes then those that did not (median 256/mm³ (IQR 189–384/mm³) vs. 111/mm³ (IQR 39–308/mm³), *p* = 0.007). Furthermore, we established significant differences in the absolute numbers of cells in the B cell subsets between patients with and without complications. (Table VI) According to the EURO class classification, two patients (4%) had less than 1% of CD19+ B cells of lymphocytes of which one patient had been diagnosed with an autoimmune complication and the other patient with a lymphoproliferative condition and gastrointestinal disease. Patients with ≥1% B cells of total lymphocytes were further divided into two categories based on the percentage of class-switched memory B cells deficiency (<2% or ≥2% of the circulating B cell pool). The percentage of patients with <2% of class switched memory B cells was 18% (11 of 46 patients). Seven of these 11 patients (63%) had one or more non-infectious complications. Patients with complications and >2% of class switched memory B cells had lower median numbers of class switched memory B cells then patients without complications. (11.2/mm³ (IQR 6.6–23.2/mm³) vs. 3.6/mm³ (IQR 0.5–10.7/mm³), *p* = 0.013).

**Table VI:** Median absolute numbers of B lymphocyte subset in CVIDs patients with and without complications

	*Total B cells*	*B cell naive*	*RBE* ^*b*^	*IgM memory*
	*CD19+N 100–400*	*CD19+sIgM+sIgD+CD10-CD27-N 72-257* ^*c*^	*CD19+sIgD+CD10+CD38+N 6-41* ^*c*^	*CD19+sIgM+sIgD+CD27+N 10-39* ^*c*^

No complications^a^ (*n* = 24)	256 (189–384)	158 (105–257)	19,5 (5–37)	30 (14–62)
Complications^a^				
Total^a^ (*n* = 28)	111 (39–308)*	71,5 (37–201)*	11,5 (5–23)	12 (2–61)
Lymphoproliverative^a^ (*n* = 16)	110,5 (16–308)*	65,5 (31–208)*	10,5 (2–18)	15,5 (2–71)
Auto immune^a^ (*n* = 13)	79 (29−139)**	60 (39−114)*	11,5 (4−21)	8,5 (2−28)*
Gastrointestinal disease^a^ (*n* = 13)	79 (22–322)*	79 (35–212)	11 (2–27)	22 (1–64)
				
	*IgG memory*	*IgA memory*	*Class switched memory B cells*	
	*CD19+sIgG+CD27+N 2-51* ^*c*^	*CD19+sIgA+CD27+N 1-20* ^*c*^	*CD19+IgD-CD27+N 2-40* ^*c*^	

No complications^a^ (*n* = 24)	7 (4–13)	3,5 (2–9)	11,5 (7–23)	
Complications^a^				
Total^a^ (*n* = 28)	1,5 (0–6)**	2 (0–6)	3,5 (0,2–11)*	
Lymphoproliverative^a^ (*n* = 16)	1 (0−7)*	2 (0−10)	3,5 (0−12)*	
Auto immune^a^ (*n* = 13)	1 (0–2)*	1,5 (0–3)*	3 (0–5)*	
Gastrointestinal disease^a^ (*n* = 13)	1 (0–11)*	3 (0–9)	4 (1–26)	

Complications: Lymphoproliverative-, Auto immun- and gastrointestinal disease. Malignancies excluded

^a^Median absolute numbers/mm³ (IQR)

^b^Recent bone marrow immigrants

^c^From: van Gent et al., Clinical Immunology (2009) 133, 95–107

* < 0,05 and ** < 0,001

**Fig. 4:**
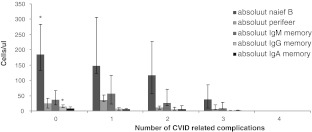
Total number of CVIDs related complications and B lymphocyte subset. * Patients with complications had significant lower number of absolute naief B cells and IgG memory B cells vs. patients without complications, *p* < 0.05

Table VI shows the median numbers within the B cell compartment and each different complication. Low numbers of switched memory B cells was associated with autoimmune and lymphoproliferative disease. Furthermore patients with splenomegaly and granulomatous disease had lower median numbers of switched memory B cells vs. patients without these conditions. (this is not shown in table 6: 9.8/mm³ (IQR 4.6–23/mm³) vs. 0.4/mm³ (IQR 0.1–3.5/mm³), *p* = 0.001, 1.5/mm³ (IQR 0−10/mm³) vs. 11.5/mm³ (IQR 7–23/mm³) *p*=0.016 respectively). Figure 4 shows the relation between the number of complications and the B cell subsets. Although not significant, there seems to be a trend of a decrease in the absolute numbers of naive B cells, as the number of complications increases. Patients with one or more complication had significant lower naïve B cells (158 vs. 71.5 cells/μl *p*=0.04) and IgG memory B cells (7 vs. 1.5 cell/ μl *p*=0.01).

#### T Cell Phenotype

T lymphocyte abnormalities were present in almost 20% of CVIDs patients (Tables V) and the majority of patients with decreased numbers of CD3, CD4 and CD8 positive T cells had been diagnosed with one or more complication, most often lymphoproliferative and autoimmune diseases. Table VII shows the median absolute numbers within the T cell compartment in patients with and without complications. Patients with one or more complications had significant lower numbers of CD3 and CD4 positive T cells. Furthermore patients with autoimmune disease had significant lower absolute numbers of CD3, CD4 and CD8 positive T cells compared to patients without complications. Absolute numbers of naive CD4+ T cells and naive CD8+ T cells were significant lower in the group of patients with any complication (Table VII).

**Table VII:** Median absolute numbers of T lymphocyte subset in CVIDs patients with and without complications

	*CD3*	*CD4*	*CD8*	*Activated CD4 T cell*	*Naive CD4 T cell*
	*(N 700–1900)*	*(N 400–1300)*	*(N 200–700)*	*(N 2,5–8,5)*	*(N 240–790)*
					
No complication^a^ (*n* = 24)	1450 (1050–1899)	812 (607–1131)	533 (372–738)	8 (3–17)	367 (223–580)
Complications^a^					
total^a^ (*n* = 28)	968 (671–1649)*	572 (330–989)*	379 (222–619)	12 (6–24)	121 (52–390)*
Lymphoproliferative^a^ (*n* = 16)	792 (643–1589)*	566 (357–1021)	270 (151–597)*	18–(9–27)	115 (14–406)*
Auto immune^a^ (*n* = 13)	806 (1050–1899)*	512 (261–578)*	335 (224–562)*	11 (5–29)	84 (43–221)*
Gastrointestinal disease^a^ (*n* = 13)	975 (560–1604)*	601 (419–989)	311 (153–558)*	11 (2–24)	99 (21–269)*

	*Eff/Mem CD4 T cell*	*Activated CD8 T cell*	*Naive CD8 T cell*	*Eff/Mem CD8 T cell*	
	*(N 150–500)*	*(N 4–19)*	*(N 220–400)*	*(N 50–190)*	
					
No complication^a^	418 (306–548)	9 (3,0–16)	308 (214–357)	233 (147–363)	
Complications^a^					
total^a^ (*n* = 28)	412 (250−569)	14 (5−25)	99 (43−156)**	237 (120−429)**	
Lymphoproliferative^a^ (*n* = 16)	507 (164–584)	20 (7–41)*	86 (30–158)**	141 (106–392)*	
Auto immune^a^ (*n* = 13)	336 (244–435)	11 (6–18)	84 (40–135)*	217 (120–495)*	
Gastrointestinal disease^a^ (*n* = 13)	513 (156–613)	7 (3–23)	64 (29–125)**	181 (106–345)*	

Complications: Lymphoproliverative-, Auto immun- and gastrointestinal disease. Malignancies excluded

^a^Median absolute numbers/mm³ (IQR)

* < 0.05 and ** < 0.001

**Fig. 5:**
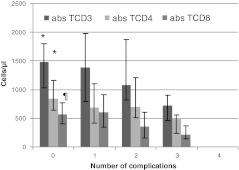
Total number of CVIDs related complications and T lymphocyte subset. * Patients with complications had significant lower number of CD3 and CD4 vs. patients without complications, *p* < 0.05. ¶ For CD8 *p* = 0.075

Figure 5 shows the relation between the number of complications and T lymphocytes subsets. The number of absolute CD3+and CD4+ T lymphocytes was significantly lower in the group of patients with one or more complication. All 50 patients that were tested on lymphocyte proliferation had normal responses on mitogens PHA, ConA and PWM (48 patients were tested) and only a few patients responded abnormal on Candida (41/45 patients, 2%), PPD (5/43 patients, (12%), tetanus toxoid (3/44 patients, 7%) and diphtheria toxin (6/40 patients, 15%)*.*

### Partial Antibody Deficiency

Of the nine patients with IgG subclass deficiency most were Caucasian (8 patients, 89%) and one patient originated from the Far East. Seven patients were female (78%) and two male (22%). The median age when symptoms started was 16 years (IQR 4-37 yrs) and the median age at diagnosis was 38 years (IQR 14-45 yrs). None of the patients died during follow up. Four patients were diagnosed with an IgG4 deficiency of which one had IgG therapy and one antibiotic prophylaxis. Two patients had an IgG2 deficiency, one on IgG therapy and antibiotics prophylaxis as well and one patient was on antibiotics only. Two patients had a IgG3 deficiency, both on antibiotic prophylaxis and one patient with a combined IgG2,3 and 4 deficiency who was treated by IgG therapy and antibiotics prophylaxis. The two patients without therapy became free of infections. One out of four patients on antibiotic prophylaxes and one on out of three patients on IgG therapy became infection free.

As shown in Table II all patients had infections prior to diagnosis which mainly consisted of respiratory tract infections. Two of the 9 patients with an IgG subclass deficiency had suffered form from bacterial meningitis. In contrast to CVID, patients with a selective IgG deficiency did not suffer from gastrointestinal infections.

Despite a median time to diagnosis of ten years (IQR 0–33 yrs). None of the patients developed chronic pulmonary disease or other complications during follow up.

Of the nine patients diagnosed with Selective antibody deficiency with normal immunoglobulins (SADNI) six were female (67%) and three male (33%) and all were Caucasian. The median age at start of the symptoms was 33 years (IQR 20–44 yrs) and the median age at diagnosis was 44 years (IQR 21–50 yrs). The median IgG at diagnosis was 10.3 g/L (IQR 6.6–17.7 g/L). None of the patients died during follow up.

Two patients were treated by immunoglobulin substitution and four patients by prophylactic antibiotics. All patients had infections (mainly respiratory tract) prior to diagnosis. Four patients without therapy, one of four patients on prophylactic antibiotics and one out of two patients on IgG therapy became free of infections. Two patients had chronic pulmonary disease at the time of diagnosis which is uncommon in SADNI patients of which one patient was diagnosed with SADNI because of the presence of bronchiectasis. This patient was started on immunoglobulin therapy and experienced fewer infections thereafter. The other patient was diagnosed with fibrosis on High resolution CT scanning of the lungs diagnosed after an episode with a complicated pneumonia. The median diagnostic delay was 6.5 years (IQR 1–30 yrs). Of the two patients with chronic pulmonary disease, only the patient with bronchiectasis had a considerable diagnostic delay (33 year).

Table VIII shows the decline in infections before and after diagnosis in the group of patients with an IgG subclass deficiency (*n* = 9) or selective antibody disorder (SADNI, *n* = 9). Immunophenotyping was performed in seven of the nine IgG subclass patients and in three of the nine SADNI patients. The median absolute numbers of B cells were normal in both groups (subclass deficiency 360/mm^3^ (IQR 170–583/mm^3^), SADNI 201/mm^3^ (IQR 165–484/mm^3^). Within the B cell compartment of IgG subclass patients the absolute median numbers of IgM memory B cells were elevated (IgM memory B cells: 54.5/mm^3^, IQR 20–111/mm^3^, ref values: 20 (10–39/mm^3^). The B cell compartment of SADNI patients showed a decreased absolute median numbers of naïve B cells and an elevated number of IgM memory B cells (naïve B cells: 61/mm^3^ IQR 54–315/mm^3^, ref values: 153/mm^3^ (72–257/mm^3^) and IgMmemory B cells: 46/mm^3^ IQR 43–56/mm^3^, ref values: 20 (10–39/mm^3^)

**Table VIII:** Number (%) of patients with infections before and after diagnosis in the group of patients with an IgG subclass deficiency (*n* = 9) or selective antibody disorder (SADNI, *n* = 9)^a^

	Before diagnosis		After diagnosis	
	Number of patients (%)		Number of patients (%)	
	IgG Subclass deficiency	SADNI	IgG Subclass deficiency^c^	SADNI^d^
Sinusitis	1 (11%)	3 (33%)	2 (22%)	2 (22%)
Otitis Media	1 (11%)	1 (11%)	1 (11%)	1 (11%)
Pneumonia	5 (56%)	3 (33%)	1 (11%)	0
Other^b^	4 (44%)	2 (22%)	2 (22%)	0

^a^Patients can have more than 1 condition

^b^Subclass deficiency: hidradenitis, meningitis, UWI. SADNI: mastitis, abdominal abcess, meningitis

^c^Two patients without therapy, four patients on antibiotic prophylaxis, one patient on IgG therapy, two patients on IgG therapy and antibiotic

^d^Four patients without therapy, three patients on antibiotic prophylaxis, one patient on IgG therapy and one patient on IgG therapy and Ab profylaxe

## Discussion

CVIDs is the most common and most diverse primary antibody deficiency. It consists of a heterogeneous group of patients with variable infectious and immunological manifestations. CVID s patients share clinical features but differ in their clinical course probably due to different underlying immunopathogenic mechanisms which are largely unknown as is the genetic cause.

This study presents the clinical and immunological data on a cohort of 61 patients. We found that the initial clinical presentation in our cohort was comparable to other cohort studies that have been published in the past [7, 29, 30]. It has been reported that 90% of CVIDs patients suffer from one or more episodes of lower respiratory tract infections prior to diagnosis [31] and our findings were compatible to that. Because of the retrospective nature of the study the exact number of infections per year could not be traced and we were only able to distinguish patients that still suffered from infections from those that did not. When these two groups were compared, median trough levels did not differ significantly. This might be due to the fact that in our clinic we aim for a IgG trough level above 8 g/L for every patient and to the fact that this cohort is too small to find differences in IgG trough levels. The overall percentage of patients affected by new respiratory infections decreased during follow-up in our cohort. However, the effect on chronic sinusitis and acute episodes of sinusitis was less impressive. Previous reports have shown that immunoglobulin therapy is effective in preventing acute respiratory infections [26, 32–36]. Structural airway changes (e.g. bronchiectasis and bronchial wall thickening), parenchymal and interstitial lesions and chronic sinusitis are common in adult CVIDs patients (38–79%) [6, 11, 31, 37–41] Despite the considerable time to diagnosis in our cohort the prevalence of symptomatic chronic pulmonary disease and chronic sinusitis at diagnosis was lower in compared to other reports (18–40% [11, 29, 31, 42, 43] and 36–90% respectively [29, 44]. In our group only 10% of the patients with chronic pulmonary disease (CPD) were found to have bronchiectasis prior to diagnosis of CVIDs, this increased to 29% with a median follow up of 7 years [4–13]. However, this is likely an underestimation as high resolution computed tomography (hRCT) has not been applied for every patient at diagnosis and during follow up. Progression to CPD continued in our cohort, despite IgG replacement therapy with adequate IgG trough levels (>8 g/L) [26] which seems to be in accordance with previous reports [29, 42, 45–47] The pathogenesis of CPD include recurrent respiratory tract infections but also non-infectious inflammatory conditions caused by immune dysregulation such as granulomatous and interstitial pulmonary disease [41] Immunoglobulin therapy has proven to be effective in preventing acute respiratory infections but conflicting data exist on the beneficiary effect on chronic pulmonary damage [11, 29, 42, 45–47] Therefore the contribution of immune dysregulation to CPD cannot be underestimated stressing the importance of early detection and directed therapy for chronic lung disease.

Chapel et al. [9] defined 5 distinct clinical phenotypes: patients with no complications, with autoimmunity, with polyclonal lymphocytic infiltration, with gastrointestinal disease or with malignancy. In our patient cohort 29% of patients had one or more complications at diagnosis, which increased to 51% of patients during follow up and despite immunoglobulin therapy. Compatible to other cohort studies [7, 29] the most common complications were autoimmune and lymphoproliferative disease. Chapel et al. described a similar frequency of 33% and 22% respectively [9]. The increase was applicable for all categories of complications, however it was most prominent in gastrointestinal disease (6.5% to 21%), described earlier [29]. Nevertheless, we established a lower prevalence of symptomatic gastrointestinal disease compared with other reports [7, 29]. Furthermore only 10% had chronic diarrhea compared 20–60% chronic diarrhea in other reports from the literature) [7, 16, 48, 49] The progression of chronic gastrointestinal diseases may still occur in patients with a primary antibody deficiency since IgG substitution will only substitute IgG, while IgA and IgM, the major secretory antibodies at mucosal surfaces are not replaced, and secondly, immune dysregulation and T cell abnormalities may contribute to gastrointestinal disease. Cytopenia was the most frequently diagnosed autoimmune disease. Three patients developed alopecia areata before diagnosis of CVIDs while alopecia in CVIDs has been described only in case reports [50–52]. Malignancies are more common in CVIDs patients at a younger age, especially gastrointestinal cancer and lymphoma [17, 18, 29] In our cohort four patients were diagnosed with cancer of which two at a really young age (thyroid cancer (22 years) and anal carcinoma (27 years)). Pulmonary and other complications continue despite adequate replacement pointing at other causes responsible for this complication. Failure to diagnose CVIDs and therefore delaying the start of adequate therapy for specific conditions can cause considerable morbidity, particularly in case of progressive airway disease. The median time to diagnosis for the CVIDs patients in our cohort (10 years) was comparable to previously reported (3–15 years) [7, 9, 10, 29, 43, 53]. In a registry of nearly 400 patients from the United Kingdom, Germany, Sweden and the Czech republic, 20% of patients were diagnosed more than 15 years after onset of symptoms [9]. In our study the mean time to diagnosis in the group of patients with one complication or more was 7 years longer (*p* < 0.05) in comparison to the group of patients without complications. Although IgG substitution therapy seemed to have little effect on the development of these or new complications, reducing the diagnostic delay is essential in order to reduce infection-related complications such as pulmonary damage and chronic sinusitis.

It must be stressed that the age at which symptoms had started is not a reliable parameter in that it is a retrospective estimation and cannot be calculated in months and variation might even be 1 or 2 years.

Different classification schemes using clinical parameters [9], flow cytometric markers of B- [13, 19, 20] and T cells [21] have been proposed in order to subdivide the heterogeneous CVIDs population into more homogenous groups which might yield clues for possible pathogenic mechanisms as well lead to a model to predict which patients are prone to complications. Recently the first genome-wide association and gene copy number variation (CNV) study in patients with CVIDs was performed which uncovered multiple novel susceptibility loci for CVIDs confirming the polygenic nature. Nevertheless these results could provide new mechanistic insights into immunopathogenesis [54].

The EURO class [13] trial established an association between a reduction in class switched memory B cells (<2%) and CVIDs related complications was associated with a higher risk for splenomegaly and granulomatous disease. In other studies this association between a reduction of peripheral switched memory B cells and other clinical complications was confirmed [13, 19, 20, 55]. In our study, most patients had been diagnosed long before the bloodsampling. However, it is generally believed that the values in T and B cell phenotyping are more or less stable during the lifecycle of the patient. To our knowledge no data is published on this subject, it has only been confirmed by word of mouth during scientific sessions. The overall incidence of patients with a low percentage (<2%) of class switched memory B cells was low. However, the association between autoimmune disease and a low percentage (<2%) of class switched memory B cells was established as well as with splenomegaly and granulomatous disease. Furthermore patients with complications and >2% of class switched memory B cells had lower median numbers of class switched memory B cells then patients without complications. Therefore, patients with complications were more affected in their number of class switched memory B cells than patients without any complicating disease. Also, patients with complications related to immune dysregulation had lower absolute numbers of CD19 positive B lymphocytes then those that did not described by Yong et al. [56]. Subsequently, we found a correlation between CD3+ T cells, CD4+ T cells, naive CD4+ and CD8+ T cells and specific complications as previous described in the literature [22]. Few studies have investigated the correlation with clinical features [22, 57]. One study found that a low count of absolute naive CD4+ T cells was associated with splenomegaly and autoimmunity. It is likely that T cells play an important role in the pathogenesis of auto inflammatory conditions in CVIDs [58–60]. According to some studies the T-cell dysregulation such as the decrease in naive CD4+ T cells in certain patients could be due to abnormal thymus function [22, 60], however accelerate T cell turnover as a result of the high infectious burden may also be an explanation [22].

In comparison, IgG subclass deficiency and SADNI patients did not develop any complications during follow-up as described in the sparse previous studies [61–63]. The effect of therapy (IgG therapy or antibiotic prophylaxis) in these patients was most prominent on the occurrences of pneumonia. Although a decline in the number of patients with infections occurred in the group of partial antibody deficiencies, no distinction could be made between patients on or off therapy. Data about the immunological and clinical profile of immunoglobulin subclass deficiency are sparse in the literature. It appears that patients with IgG1 and/or IgG3 deficiency are more likely to have chronic and recurrent infections of the lower airways, while those with IgG2 and/or IgG4 deficiency are more likely to suffer from sinusitis and otitis [64]. Interestingly, in our cohort two of 9 patients had suffered from bacterial meningitis, which to our knowledge has not been described in earlier publications.

In conclusion, in our study the spectrum of illness for patients with CVIDs is in concordance with previous reports with predominantly respiratory tract infections prior to diagnosis. Also, infections diminished considerably as a result of IgG therapy, although this effect was considerably less for acute and chronic sinusitis. Second, the development of chronic pulmonary disease non-infection related complications was not halted by adequate immunoglobulin therapy. A considerable number of CVIDs patients already had complications at the time of diagnosis and this number of patients increased despite immunoglobulin therapy. In our cohort an association between immunological parameters and the specific complications related to CVIDs could be established within the B and T cell compartment.

The time to diagnosis in the group of patients with complications was significantly longer comparable to the group of patients without complications, and especially patients presenting with autoimmune phenomena are often under diagnosed. It remains important to increase awareness among doctors for the variable clinical presentations and manifestations of CVIDs. Specific disease related therapy would be started in an earlier stage of the disease which could affect morbidity and mortality.

## Electronic supplementary material

Below is the link to the electronic supplementary material.

ESM 1 (PDF 56 kb)
