# Supplementary material for: The Spectrum of Disease Manifestations in Patients with Common Variable Immunodeficiency Disorders and Partial Antibody Deficiency in a University Hospital
Source: J Clin Immunol. 2012 Apr 13;32(5):907–21. doi: 10.1007/s10875-012-9671-6 (PMC3443482; doi:10.1007/s10875-012-9671-6)
Supplement: Supplementary file 1 — (PDF 56 kb) [file 10875_2012_9671_MOESM1_ESM.pdf]

Springer

the language of science

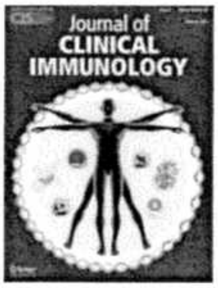

Conflict of Interest Disclosure Form

It is the policy of *Journal of Clinical Immunology* to ensure balance, independence, objectivity, and scientific rigor in the Journal. All authors are expected to disclose to the readers any real or apparent conflict(s) of interest that may have a direct bearing on the subject matter of the article. This pertains to relationships with pharmaceutical companies, biomedical device manufacturers or other corporation whose products or services may be related to the subject matter of the article or who have sponsored the study.

The intent of the policy is not to prevent authors with potential conflict of interest from publication. It is merely intended that any potential conflict should be identified openly so that the readers may form their own judgements about the article with the full disclosure of the facts. It is for the readers to determine whether the authors' outside interest may reflect a possible bias in either the exposition of the conclusions presented.

The corresponding author will complete and submit this form on behalf of all authors listed below.

Article Title The spectrum of illnesses in patients with common variable immunodeficiency in a university hospital.

Authors L.J. Maarschalk, Prof. Dr. A.I.M. Hoepelman, J.M. van Montfrans, P.M. Ellerbroek.

MS Id. No. ……………………………………………

Please note that a conflict of interest statement is published with each paper.

I certify that there is no actual or potential conflict of interest in relation to this article. If any conflict exists, please define hereafter:

Conflict (if none, "None" or describe financial interest/arrangement with one or more organizations that could be perceived as a real or apparent conflict of interest in the context on the subject of this article):

None

Name L.J. Maarschalk - Ellerbroek

Signature ………………………… Date 22-12-2011

Please scan the completed and signed form and then submit it as an electronic file along with your manuscript. No manuscripts will be accepted for review without this form.

Springer
http://www.springer.com/journal/10875
Journal of Clinical Immunology
Editor-in-Chief: Thomas A. Fleisher
Deputy Editor-in-Chief: Kathleen Sullivan
ISSN: 0271-9142 (print version)
ISSN: 1573-2592 (electronic version)
Journal no. 10875
Springer
